# COVID-19: Modeling Out-of-Home and In-Home Activity Participation during the Pandemic

**DOI:** 10.1177/03611981211067790

**Published:** 2022-01-22

**Authors:** Md. Shahadat Hossain, Khademul Haque, Mahmudur Rahman Fatmi

**Affiliations:** 1 School of Engineering, Civil Engineering, University of British Columbia, Okanagan Campus, Kelowna, BC, Canada; 2 Transportation Planner, CDM Smith Inc., Austin, TX

**Keywords:** planning and analysis, traveler behavior and values, activity, activity-based modeling, behavior analysis, behaviors, general, pattern (behavior, choices etc.)

## Abstract

Understanding the interaction between in-home and out-of-home activity participation decisions is important, particularly at a time when opportunities for out-of-home activities such as shopping, entertainment, and so forth are limited because of the COVID-19 pandemic. The travel restrictions imposed as a result of the pandemic have had a massive impact on out-of-home activities and have changed in-home activities as well. This study investigates in-home and out-of-home activity participation during the COVID-19 pandemic. Data comes from the COVID-19 Survey for assessing Travel impact (COST), conducted from March to May in 2020. This study uses data for the Okanagan region of British Columbia, Canada to develop the following two models: a random parameter multinomial logit (RPMNL) model for out-of-home activity participation and a hazard-based random parameter duration (HRPD) model for in-home activity participation. The model results suggest that significant interactions exist between out-of-home and in-home activities. For example, a higher frequency of out-of-home work-related travel is more likely to result in a shorter duration of in-home work activities. Similarly, a longer duration of in-home leisure activities might yield a lower likelihood for recreational travel. Health care workers are more likely to engage in work-related travel and less likely to participate in personal and household maintenance activities at home. The model confirms heterogeneity among the individuals. For instance, a shorter duration of in-home online shopping yields a higher probability for participation in out-of-home shopping activity. This variable shows significant heterogeneity with a large standard deviation, which reveals that sizable variation exists for this variable.

COVID-19 (also known as coronavirus) has made a significant impact on our daily life. The outbreak started in December 2019 in Wuhan, China and rapidly spread to many countries all over the world. In March 2020, the World Health Organization (WHO) declared the outbreak to be a pandemic, with countries such as China, Italy, Spain, and the U.S. being hit the hardest. As of April 14th, 2020, more than 1,750,000 people had been infected and more than 110,000 had died from the virus (*1*). Numerous countries have taken unprecedented measures to prevent social contact and to slow down the spread of the virus, such as closing schools, shops, restaurants, and bars, prohibiting public events, and stimulating or imposing working-from-home. These measures can all be labeled “social distancing” and are especially efficient for diseases (such as COVID-19) which are transmitted by respiratory droplets and require certain proximity of people (*1*). Travel restrictions were imposed around the world; for example, in late March, Canada closed its border coupled with the implementation of local travel restrictions within the country. As a result, there was a massive impact on out-of-home activities which resulted in increased duration of in-home activities.

Out-of-home and in-home activities are interdependent decisions (*2*). According to the activity-based modeling approach, the demand for travel is derived from the need to participate in activities distributed in space and time (*3*). In an activity-based framework, the two main types of activity that lead to travel decisions are out-of-home activity and in-home activity. Travel choices are mainly a result of the focus on out-of-home activity episodes whereas in-home activity episodes are not adequately analyzed in these frameworks. In-home and out-of-home activity episodes have quite different implications for travel: an in-home episode does not involve travel (for a person already at home), while an out-of-home episode requires travel (*4*).

There are several relevant studies that have contributed to the literature of out-of-home activity participation modeling before the world was hit by the COVID-19 pandemic. Born et al. (*5*) adopted a copula-based joint generalized extreme value (GEV) duration modeling technique to jointly model weekend discretionary activity participation and episode duration. The study reveals the impact of socio-demographic attributes—for example, age, income, gender, and so forth—and household location on both discretionary activity participation and episode duration. Chu (*6*) investigated workers’ maintenance activity participation and duration over different time periods in a working day. The model results confirm the effect of travel characteristics such as commute time and mode, duration of work, and so forth on the activity participation and duration of the workers. Built-environment attributes such as employment density and location of both work and home are also found to significantly affect the activity participation and duration of the workers. In addition, the findings also reveal the effect of socio-demographic characteristics such as age, income, and gender on maintenance activity participation and duration. Feng et al. (*7*) investigated the intra-personal and inter-personal interactions of partners/spouses and inter-personal interactions between different generations in elderly co-residence households. Habib and Daisy (*8*) investigated the frequency and the duration of participation in physical activity by school-going children. Kizony et al. (*9*) proposed a model that explains the activity participation of community-dwelling older adults. The model examines travel attitudes and mobility behaviors as mediating factors between personal characteristics and participation in out-of-home daily activities. Spinney et al. (*10*) investigated the effect of having a driver’s license on out-of-home and social activity engagement and duration among non-working older (≥65 years) Canadians. They also investigated the distribution of these impacts across socio-demographic and self-reported health domains. Spissu et al. (*11*) presented the analysis and modeling of weekly activity-travel behavior using a unique multi-week activity-travel behavior data set and a panel version of the Mixed Multiple Discrete Continuous Extreme Value (MMDCEV) model. The findings of the study suggest that individual-level attributes such as age, income, employment status, occupation, and so forth, and household demographics such as residential location significantly affect out-of-home discretionary activity engagement.

Very few studies were conducted on in-home activity participation. Termida and Susilo (*12*) examined the effects of out-of-home and in-home constraints such as working-from-home, studying at home, and doing household maintenance activities on individuals’ day-to-day leisure activity participation decisions in four different seasons using dynamic ordered probit models. The study found that individuals spending longer duration in work-related activities are less likely to participate in leisure activities regardless of the season. Susilo et al. (*13*) investigated the long-term trends in activity travel patterns of individuals in different life-cycle stages and generations.

Some of the studies contributed toward the out-of-home and in-home activity interactions. Bhat and Gossen (*4*) presented a mixed multinomial logit model for the type of recreational activity episodes that individuals pursue during the weekend. The type of recreational episode includes in-home and out-of-home episodes. Dharmowijoyo et al. (*14*) investigated the interaction among individuals’ non-instrumental variables, time-space (such as their day-to-day time duration of activity participation, socio-demographics, and built environment), and health factors on individuals’ day-to-day discretionary activities. Mosa and El Esawey (*3*) showcased an empirical investigation of household interactions in joint in-home and out-of-home daily maintenance activity participation. A statistically significant and highly negative value of error correlation value suggests that the unobserved factors inversely contribute to the in-home and out-of-home maintenance activity participation. The analysis reveals the significant effect of socio-demographic characteristics (e.g., age, income, household composition, employment, etc.) and built-environment variables among others on both in-home and out-of-home activity participation.

Srinivasan and Bhat (*2*) investigated the in-home maintenance activity generation of single-worker and dual-worker households with children for both male and female heads. They found that out-of-home work duration is negatively associated with in-home activity generation for both types of households. Shabanpour et al. (*15*) jointly modeled in-home activity type and duration in an ADAPTS modeling framework and found direct effects of different types of out-of-home activity duration on different in-home activity participation and duration. For instance, individuals who work for a longer duration out-of-home are less likely to participate in leisure and discretionary activities at home. Longer duration of out-of-home household maintenance activities may result in a shorter duration of personal maintenance at home (*15*). Dharmowijoyo et al. (*16*) examined the interdependencies among an individual’s time allocation for different activities and other parameters. These parameters include travel time spent on a given day and socio-demographic and built-environment variables. The variables comprise time duration of in-home and out-of-home discretionary activities, and how the interaction of these variables influences an individual’s activity space indices on the time duration of discretionary activities.

Looking at the effect of mobility tool ownership on activity participation, research is very limited. Mobility tools refer to tools that give access to a travel mode such as driver’s license, transit pass, rideshare subscription, personal vehicle, and so forth (*17*, *18*). Because of the travel restrictions imposed during the pandemic, mobility tool ownership might have a significant effect on individuals’ activity participation out-of-home. Since the onset of the COVID-19 pandemic, very few studies were conducted to analyze the impact of COVID-19 on activity participation. De Vos (*1*) discussed the potential implications of social distancing on daily travel patterns. He concluded that avoiding social contact might completely change the number and types of out-of-home activities people perform, and how people reach these activities. He also mentions that the demand for travel is expected to be reduced and that people will travel less by public transport. In another study, Cartenì et al. (*19*) quantified the effect of mobility habits in the spread of the Coronavirus in Italy. The key findings are that mobility habits explain the number of COVID-19 infections jointly with the number of tests/day and some environmental variables, such as the areas close to the outbreak having a higher risk of contagion, especially in the initial stage of infection. Furthermore, the number of daily new cases was related to the trips performed three weeks before.

Based on the literature review, it can be identified that a gap in the literature exists with respect to understanding the impact of travel restrictions on in-home and out-of-home activity participation decisions, particularly at a time when opportunities for out-of-home activities such as shopping, entertainment, and so forth are limited because of the COVID-19 pandemic. Since the onset of the COVID-19 pandemic is still very recent, on a limited number of studies have examined how individuals are adjusting their in-home and out-of-home activities as a result of the novelty of COVID-19. Therefore, the main contribution of this paper is to investigate individuals’ daily out-of-home and in-home activity participation during the COVID-19 pandemic. One of the key features of this study is to examine the interactions between in-home and out-of-home activities. The interactions are explored by broadly examining: (i) how in-home activity affects the participation in out-of-home activities; and (ii) how out-of-home activity participation affects the in-home activities. Several hypotheses were tested to reveal the interactions. For instance, it is hypothesized that a higher frequency of work-related out-of-home activities might lead to a lower duration of in-home activities. Similarly, a higher duration of in-home leisure activity is hypothesized to be negatively associated with participation in out-of-home recreational or social activities. This study further tested several hypotheses in relation to individuals’ socio-demographics and travel characteristics. Methodologically, out-of-home activity participation is modeled adopting a random parameter multinomial logit (RPMNL) modeling technique. In the case of the in-home activity, the duration of activity participation is modeled adopting a hazard-based random parameter duration (HRPD) method. The purpose of adopting the random parameters extension of the logit and hazard models is to capture unobserved heterogeneity among the individuals.

## Data

Data for this study come from the COVID-19 Survey for assessing Travel impact (COST) for the Okanagan region of British Columbia in Canada. COST was an online-based survey conducted from March 24 to May 9, 2020. The focus of this survey was to collect information with regard to individuals’ immediate responses to COVID-19, focusing on adjustments to their daily activity behavior. The survey was distributed using a convenience sampling technique. The publicity for the survey was done by social media advertising on platforms such as Facebook and Twitter and by posting in public groups; incentives were provided to the respondents through a random draw for participating in the survey. On March 17, 2020, social distancing measures were imposed in British Columbia, accompanied by the declaration of the public health emergency the next day (*20*). During this time, any social gatherings of more than 50 people were banned or temporarily shut down, while the opening hours of major businesses including restaurants and pubs were limited. All dine-in food services were prohibited; only takeout options were allowed. Educational institutions were closed with classes moving online. The numbers of passengers permitted in public transit were limited. However, there were no punitive measures imposed on people for not staying at home.

The survey collected information with regard to daily activity, long-distance travel, and socio-demographic characteristics during the COVID-19 pandemic. Daily activity has two components: in-home and out-of-home activities. The in-home activity component collected information on the frequency and duration of different activities for an entire day, specifically during the most recent weekday. The in-home activities can be categorized as: sleep which includes night sleep and daytime naps; personal maintenance which includes personal care, eating drinking, grooming, and so forth; household maintenance which includes general household activity, house cleaning, and caring for household members; leisure activities which include relaxing, socializing, watching TV, exercise, hobbies, or games; discretionary activities which include religious or spiritual and volunteer activity; mandatory activities which include work or school-related activity; online shopping for groceries and medical supply; online shopping for other products such as food from restaurants, clothing; and other activities. The question was presented to the respondents as, “During the most recent weekday, provide information on the duration and frequency of each in-home activity that was performed.”

The out-of-home activities can be thematically categorized into the following activity types: work-related which includes work or work-related errands and school-related work; household errands which include personal business, household errands, shopping for major purchases, picking up or dropping off passengers, and health care; shopping activities; recreational/social which includes civic or religious activities, recreation, and visiting friends or family; and picking up online orders of groceries, medical supplies, food from a restaurant, clothing; household maintenance; and so forth. The out-of-home activity component primarily asked the respondents about their participation in different out-of-home activities for an entire day, specifically during the most recent weekday. This question was asked in a binary form of yes or no. This activity participation information is used to form a choice set that includes the following activity types: work-related, household errands and others, picking up online orders, shopping, and recreational/social. The above discrete activity types are used as the dependent variable in the out-of-home activity participation model. Further information on the frequency, companionship, travel satisfaction and happiness, and travel mode while participating in different types of out-of-home travel activities on that day were collected. Finally, the socio-demographic component collected information on respondents’ age, income, gender, marital status, employment status, level of education, number of adults and children in the household, tenure type, dwelling type, vehicle ownership, and whether they had a driver’s license.

**Figure 1.**
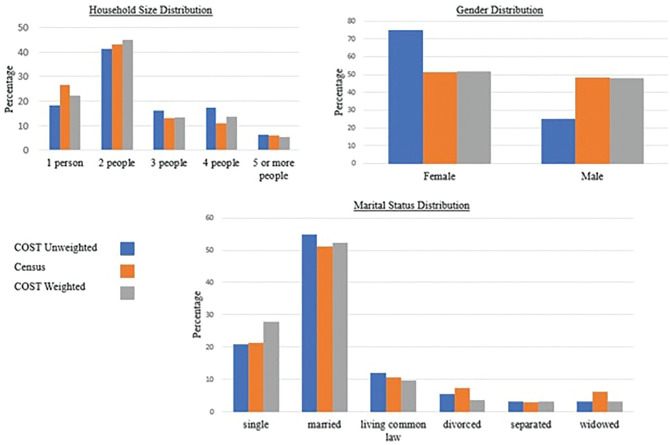
Comparison of gender, household size, and marital status distributions in COST data, census statistics, and the weighted sample. *Note*: COST = COVID-19 Survey for assessing Travel impact.

The survey used a convenience sampling technique. The respondents were not randomly selected. Some attributes of the sample did not represent the Okanagan population according to the 2016 Census statistics of Canada. For instance, the survey data under-represented the male population by 23.54%, where the distributions for males in the survey data and census were 25% and 48.54%, respectively. In the case of marital status, the distributions for married individuals were 54.95% and 51.28% in the survey data and census statistics, respectively. Therefore, the sample was weighted to represent the characteristics of the Okanagan population. In the case of the weighting technique, an iterative proportional fitting (IPF) approach was adopted (*21*). During weighting, age and income were considered as the control variables (*22*). The validation results suggest that the weighted sample reasonably represents the Okanagan population, where the majority of the categories of the attributes from the survey lie within a few percentage points from the census data. For example, the distributions of males in the weighted sample and census statistics were 48.02% and 48.54%, respectively, which indicates that the sample under-represented the population by only 0.52%. Similarly, the weighted sample over-represented married individuals by only 1.05%. The comparison of the distribution for different variables in the unweighted survey data, census population, and weighted sample is represented in Figure 1. Further description of the weighting technique and validation results can be found in Fatmi et al. (*23*). After cleaning the data for missing information, and applying weights and validation, the complete data set includes 202 responses. In total, 272 out-of-home travel activities were recorded, among which the proportion of travel activities for work, household errands, shopping, recreational/social, and picking up online orders are 18.01%, 10.29%, 36.76%, 16.18%, and 18.75%, respectively. In the case of in-home activities, 829 in-home activities were recorded, among which the distribution of sleep, personal maintenance, household maintenance, leisure activities, discretionary activities, mandatory, online shopping for groceries and medical supplies, online shopping for other products such as food from restaurant and clothing, and other activities are 19.90%, 20.27%, 17.37%, 19.30%, 3.26%, 9.65%, 2.05%, 4.46%, and 3.74%, respectively. Although the sample substantially represents the population, it significantly over-represents car owners which is evident from the higher average number of vehicles in the household (3.03).

## Methodology

### Out-of-Home Activity Participation Model

In response to the imposed COVID-19 travel restrictions, individuals significantly reduced the number of trips they made per day to participate in different out-of-home activities. For example, the number of trips per day by an individual was found to have dropped by more than 50%. Many individuals did travel to perform different activities. For example, the COST survey revealed that, on average, around 1.32 out-of-home travel activities were performed by a person in a day. Many studies revealed a significant change in out-of-home travel activities where the majority of the trips were made for shopping purposes rather than for work or other activities (*24*). As a result, several studies attempted to understand individuals’ involvement in different travel activities such as shopping, eating-out, and leisure (*24* [Bibr bibr25-03611981211067790]–*26*). In this line of research, a holistic approach is required to examine individuals’ choices among different out-of-home travel activities during the pandemic, which is a discrete choice scenario. Therefore, this study has adopted a discrete choice modeling technique to investigate participation in out-of-home travel activity.

Since capturing unobserved heterogeneity was one of the motivations for this study, a RPMNL model, also known as the mixed logit model, is developed to investigate the factors affecting individuals’ participation in out-of-home activities. The RPMNL model is based on the random utility-based discrete choice modeling technique where an individual chooses the alternative that maximizes the utility. The general form of the utility function for choosing a particular type of out-of-home activity is given by:

(1) $$U_{\mathrm{ij}}=\beta \prime x_{\mathrm{ij}}+\varepsilon_{j}$$

where, $U_{\mathrm{ij}}$ is the utility derived by an individual *i* for an activity type *j*, and β′ is the parameter associated with explanatory variable vector $x_{\mathrm{ij}}$ . Vector $\varepsilon_{j}$ is the random error term which follows the assumption that it is independently and identically distributed (IID) across the individuals with a mean of zero, a variance σ^2^, and that it follows Type-I Extreme Value (EV) distribution (*27*). The conditional probability $P_{\mathrm{ij}}$ of an individual *i* participating in a particular type of activity *j* (*j = *1, 2, …, *J*) is represented by the following equation:

(2) $$P_{\mathrm{ij}}=\frac{e^{\beta \prime x_{\mathrm{ij}}}}{\sum_{j=1}^{J}\beta \prime x_{\mathrm{ij}}}$$

The unconditional probability can be calculated by the following equation:

(3) $$P_{\mathrm{ij}}(\beta \prime )=\int^{\;}\frac{e^{\beta \prime x_{\mathrm{ij}}}}{\sum_{j=1}^{J}\beta \prime x_{\mathrm{ij}}}f(\beta \prime |\mu ,\sigma )d\beta_{i}$$

Here, f(β′|μ,σ) is the probability density function which is assumed to be normally distributed with a mean μ and variance σ . The log likelihood function is developed based on Equation 3 to estimate the parameter values as follows:

(4) $$\mathrm{LL}=\sum_{j=1}^{J}\int \frac{e^{\beta \prime x_{\mathrm{ij}}}}{\sum_{j=1}^{J}\beta \prime x_{\mathrm{ij}}}f(\beta \prime |\mu ,\sigma )d\beta_{i}$$

There is no analytic solution for the above log likelihood equation as it is in closed form. Therefore, the log likelihood function is maximized using Quasi-Monte Carlo (QMC) simulation using 200 Halton draws (*4*). The simulated log likelihood function is represented as follows:

(5) $$\mathrm{SLL}=\sum_{j=1}^{J}\ln\frac{1}{K}\sum_{k=1}^{K}P_{\mathrm{ij}}({\beta \prime }^{k})$$

where, K is the total number of draws.

### In-Home Activity Duration Model

In the case of in-home activities, the majority of the population were staying home at the beginning of the pandemic, and they were engaged in a wide range of activities at home. Therefore, it was important to examine how individuals allocated their time for different in-home activities. For instance, time spent working-from-home has increased by more than 60 min per day in 40% of cases. Leisure activities at home have been increased by more than 60 min per day in around 85% of cases. Similar trends are observed in the case of household maintenance activity which has increased by around 60 min for 60% of cases. Therefore, this study examines the length of time individuals were involved in different activities at home, which is a continuous variable. As a result, a hazard-based duration modeling technique is adopted for the in-home activity model.

The study develops a HRPD model to determine the factors affecting the duration of in-home activities. For duration analysis, the Hazard-based duration (HDR) modeling technique is more appropriate, since the duration itself has an impact on the termination probability of the event (*28*, *29*). To capture the effects of such dynamics of duration on the probability of terminating an in-home activity, the HDR model is adopted (*30*, *31*). The traditional HDR model is extended to a random parameter HDR model with the motivation to address the unobserved heterogeneity. This model captures heterogeneity by allowing a continuous distribution of parameters among the sample individuals. The model considers the duration of a particular in-home activity as a non-negative continuous dependent variable. It is assumed that the length of time has a cumulative distribution function F(t) and a probability distribution function f(t) . F(t) is also known as failure function which is the probability of the duration of an in-home activity being less than a specified time t (*32*). On the other hand, survival function S(t) is the probability of the activity being greater than t . The relationship can be represented as follows:

(6) F(t)=P(T≤t)=1−P(T>t)=1−S(t).

The hazard function for an activity of duration *t* can be represented as follows:

(7) $$h\left(t\right)=P\left(T\leq t\right)=\lim_{\Delta t\to 0}\frac{P(t\leq T\leq t+\Delta t|T\geq t)}{\Delta t}=\frac{f(t)}{S(t)}.$$

Here, P(t≤T≤t+Δt|T≥t) represents the probability that an individual will end the activity within time *t* and *t*+ Δ *t*. Some of the widely used parametric distributions of the hazard functions are Weibull, log-logistic, and lognormal distributions. The study considers Weibull distribution as the HRPD model as this distribution provides a better model fit and is widely used for duration modeling (*33*, *34*). The hazard function takes the following form in Weibull distribution:

(8) $$h\left(t\right)=\omega \lambda (\lambda t{)}^{\omega -1}\omega ,\;\lambda >0$$

where, λ is the scale parameter and ω is the shape parameter. Since the study considers the effects of different explanatory attributes in addition to the effect of duration, the hazard function takes the following form:

(9) $$h\left(t|x\right)=h_{0}(te^{\beta \prime x})e^{\beta \prime x}$$

where,

$h_{0}$ is the baseline hazard function,

β′ is the parameter associated with the explanatory variables, and

x is the vector of explanatory variables.

An accelerated failure time (AFT) approach is used in the model for ease of interpretation. To capture the unobserved heterogeneity using the random parameters, the estimable parameters can be represented as:

(10) $$\beta_{j}=\beta +\omega_{j}$$

where, $\beta_{j}$ is the vector of parameters, and $\omega_{j}$ is error term which is assumed to be normally distributed with mean zero and variance $\sigma^{2}$ (*32*, *34*). Finally, the hazard function takes the following form:

(11) $$h\left(t|x,\omega_{j}\right)=h_{0}(te^{\beta_{j}\prime x})e^{\beta_{j}\prime x}$$

A simulated log likelihood approach is taken to determine the parameter value from the above Equation 11. A QMC simulation technique is used, using 200 Halton draws to maximize the simulated log likelihood. It was found that 200 Halton draws are considered sufficient for better parameter estimation (*33*). The “NLOGIT Version 6” econometric software package was used to estimate both RPMNL and HRPD models.

## Model Results

The study tested a wide variety of variables in both of the models which can be categorized as socio-demographic characteristics, travel characteristics, in-home activity duration, and variables representing the interaction between socio-demographics and different types of out-of-home activity participation. The socio-demographic characteristics included age, income, gender, level of education, employment status, dwelling type, and so forth. Travel characteristics variables included mobility tools such as having a driver’s license, transit pass, vehicles, rideshare subscription, and so forth, and travel companionship. The study also tested the effect of interaction between different age, gender, and occupation groups, and different types of out-of-home activity participation on in-home activity duration. We have tested multicollinearity among the variables retained in the final model using variance inflation factor (VIF) (*11*). In the case of VIF, values ranging from 0 to 5 represent no significant collinearity and when the value exceeds 5, significant collinearity exists between the two variables (*11*). The VIF estimation results confirm that no such collinearity exists among the variables retained in the model, as the maximum VIF value was found to be 2.25.

### Out-of-Home Activity Participation Model Results

The summary statistics of the variables retained in the out-of-home activity participation model are represented in Table 1. In the survey, information on the employment status was collected in the following thematic categories: unemployed, full-time worker, part-time worker, self-employed, student full-time, student part-time, retired, homemaker, volunteer, and others. Therefore, in the case of the dummy variable representing full-time workers, all other categories including unemployed are assumed as reference. In the out-of-home activity participation model, all employment status except full-time worker is held as reference for full-time workers. Similarly, the survey collected occupation information in the following categories: management; natural and applied sciences and related; health, education, law and social, community and government services; art, culture, recreation, and sport-related; sales, and service occupations; trades, transport and equipment operators and related; and others. Therefore, in the case of a dummy variable representing individuals in health care, management, and a sales occupation, all employed individuals except these three occupations are assumed as reference.

**Table 1.** Summary Statistics of the Variables Retained in Out-of-Home Activity Participation Model

Variables	Description	Mean or percentage	Standard deviation
Socio-demographic characteristics			
Age 65 or more (reference)	Individual’s age is 65 or more (dummy)	30.88%	na
Age 18–34	Individual’s age is between 18 and 34 (dummy)	18.38%	na
Age 35–64	Individual’s age is between 35 and 64 (dummy)	50.74%	na
Income $50,001–$79,999 (reference)	Individual’s yearly income is $50,001–$79,999 (dummy)	22.43%	na
Income ≤ $50,000	Individual’s yearly income is ≤ $50,000 (dummy)	23.90%	na
Income > $80,000	Individual’s yearly income is > $80,000 (dummy)	53.68%	na
Female (reference)	Individual is female (dummy)	52.21%	na
Male	Individual is male (dummy)	47.79%	na
Non-full-time worker (reference)	Individual is not a full-time worker (including unemployed) (dummy)	58.82%	na
Full-time worker	Individual is a full-time worker (dummy)	41.18%	na
Occupation: other (reference)	Individual is not a health/management/sales professional (dummy)	61.40%	na
Occupation: health	Individual is a health care professional (dummy)	8.82%	na
Occupation: management	Individual is a business, finance, and administration professional (dummy)	25.37%	na
Occupation: sales	Individual is a sales and service professional (dummy)	4.41%	na
Travel characteristics			
Does not hold a driver’s license (reference)	Individual does not hold a driver’s license (dummy)	4.78%	na
Holds a driver’s license	Individual holds a driver’s license (dummy)	95.22%	na
Vehicle ownership	Number of vehicles in the household	3.03	1.22
Travel companionship: other (reference)	Does not travel with partner or children while performing the out-of-home activity (dummy)	83.09%	na
Travel companionship: family	Travels with partner or children while performing the out-of-home activity (dummy)	16.91%	na
In-home activity duration			
Mandatory duration above average (reference)	Mandatory in-home activity duration above average (average duration 351.79 min) (dummy)	27.21%	na
Mandatory duration below average	Mandatory in-home activity duration below average (average duration 351.79 min) (dummy)	72.79%	na
Online grocery shopping above average (reference)	Online shopping duration for groceries above average (average duration = 27.6 min) (dummy)	4.78%	na
Online grocery shopping below average	Online shopping duration for groceries below average (average duration = 27.6 min) (dummy)	95.22%	na
Leisure activity below average (reference)	In-home leisure activity duration below average (average duration = 229.94 min) (dummy)	67.65%	na
Leisure activity above average	In-home leisure activity duration above average (average duration = 229.94 min) (dummy)	32.35%	na
Discretionary activity below average (reference)	In-home discretionary activity duration below average (average duration = 60.86 min) (dummy)	24.63%	na
Discretionary activity above average	In-home discretionary activity duration above average (average duration = 60.86 min) (dummy)	75.37%	na

*Note:* na = not available.

The parameter estimation results and goodness-of-fit measures of the out-of-home activity participation model are represented in Table 2. The adjusted pseudo r-squared, Akaike Information Criterion (AIC), Bayesian Information Criterion (BIC) values of the model are 0.26, 720.5, and 846.70, respectively. To compare the goodness-of-fit measures, a traditional multinomial logit (MNL) model was developed. The MNL model yields a lower adjusted pseudo r-squared value (0.16) than the RPMNL model but a higher AIC (736.20) and BIC (847.90) value. The comparison indicates that the RPMNL model ensures a better goodness-of-fit than the MNL. Besides, some of the important variables such as attitude, lifestyle, built environment, and so forth were not tested in the model which might be captured in the randomness of the new parameter and thus provide a better model fit. For further comparison between the models, a likelihood ratio test is performed according to Bhat and Gossen (*4*). The likelihood ratio is found to be 225.02 which is significantly greater than the critical chi-squared value for three degrees of freedom. Therefore, the presence of unobserved heterogeneity cannot be rejected and the RPMNL model is considered as the final model to investigate the out-of-home activity participation decisions.

**Table 2.** Parameter Estimation Results of the Out-of-Home Activity Participation Model

Variables	Work-related		Household errands and others		Picking up online orders		Shopping		Recreational/social	
	Coef.	t-stat.	Coef.	t-stat.	Coef.	t-stat.	Coef.	t-stat.	Coef.	t-stat.
Constant	na	na	2.95	1.54	1.94	0.93	1.79	0.77	1.88	1.53
Socio-demographic characteristics										
Age 18–34	na	na	−5.09***	−3.04	−4.86***	−3.20	−5.57***	−3.04	na	na
Age 35–64	2.20*	1.93	na	na	na	na	na	na	na	na
Income > $80,000	−3.49**	−2.20	0.89	1.13	na	na	na	na	−15.22	−1.52
Income ≤ $50,000	na	na	na	na	3.02***	3.46	2.15**	2.37	na	na
Gender: male	na	na	0.97*	1.72	na	na	na	na	na	na
Full-time worker	2.65**	2.09	na	na	na	na	na	na	−1.62	−1.37
Occupation: health	1.78	1.15	na	na	−1.97*	−1.68	na	na	na	na
Occupation: management	na	na	0.81	1.26	na	na	na	na	na	na
Occupation: sales	na	na	3.16***	2.79	na	na	na	na	na	na
Travel characteristics										
Holds a driver’s license	na	na	na	na	2.60**	2.03	3.35**	2.07	na	na
Vehicle ownership	0.85**	2.19	0.40	1.03	0.31	1.00	na	na	na	na
Travel companionship: family	na	na	na	na	2.05***	2.90	1.46*	1.88	na	na
In-home Activity duration										
Mandatory duration below average	2.02**	2.08	na	na	na	na	na	na	na	na
Online grocery shopping below average	na	na	na	na	na	na	1.53*	1.89		
Leisure activity above average	na	na	na	na	na	na	na	na	−3.00***	−3.14
Discretionary activity above average	na	na	na	na	na	na	na	na	5.63***	3.04
Standard deviation of the random parameters										
Income > $80,000	4.22**	2.16	na	na	na	na	na	na	17.31*	1.78
Online grocery shopping below average	na	na	na	na	na	na	2.04	1.43	na	na
Goodness-of-fit measures										
Log likelihood function										−325.26
Adjusted pseudo r-squared										0.26
AIC										720.5
BIC										846.70

*Note*: Coef. = coefficient; t-stat. = t-statistic; na = not significant; AIC = Akaike Information Criterion; BIC = Bayesian Information Criterion; *** = 1% significance; ** = 5% significance; and * = 10% significance.

The model results reveal that individuals’ out-of-home activity participation during the COVID-19 pandemic was significantly affected by their socio-demographic attributes, travel behavior, and in-home activity duration. Among the socio-demographics, an individual’s age, yearly income, gender, employment status, and occupation are found to have significant effect on their preferences for participating in different out-of-home activities. Younger individuals (aged from 18 to 34) are less likely to participate in household errands (personal business, major shopping, picking up or dropping off passengers), picking up online orders (groceries, medicine, food, restaurants, etc.), and regular out-of-home shopping activities. Middle-aged individuals (aged from 35 to 64) are more likely to perform work-related out-of-home activities. Individuals with higher income (yearly income > $80,000) are less likely to perform work-related activities whereas they have a higher likelihood of participating in household errands. These results indicate that the need for work-related out-of-home activities has reduced because of the pandemic as many of the businesses and workstations have moved online to ensure safety and to reduce the spread of the virus. Such individuals may, therefore, use this opportunity to perform household errands. However, significant heterogeneity can be observed from the statistically significant and higher value of standard deviation which indicates that they may also perform work-related out-of-home activities. In addition, high-income individuals are less likely to perform out-of-home recreational activities (e.g., entertainment, visit a friend, and civic or religious activities) although heterogeneity exists among the sample individuals which is evident from the statistically significant and higher value of standard deviation of the random parameter. Individuals with a lower income (yearly income ≤ $50,000) are still likely to travel outside to pick up online orders and for shopping. Male individuals have a higher likelihood of performing household errands. Further, full-time workers are more likely to perform work-related activities. Health care professionals are the front-line workers fighting the pandemic and the model results suggest that they are more likely to participate in work-related out-of-home activities. On the other hand, they are less likely to travel to pick up online orders as they might have been too busy providing health care services. Other professionals such as individuals in management and sales services have a higher likelihood of performing household errands.

Individuals’ travel characteristics also affect their participation in out-of-home activities which is evident from the model results. Holding a driver’s license is found to positively affect participation in picking up online orders and shopping activities. Individuals living in a household with a higher number of vehicles have a higher likelihood of performing work-related, household errands and other activities, such as picking up online orders. These results show that owning a mobility tool significantly affects an individuals’ out-of-home activity participation during the pandemic as it will enable them to travel out-of-home with minimal in-person contact and ensure more safety from getting infected.

One of the major findings of this study is the effect of in-home activity duration on individuals’ out-of-home activity participation. Individuals performing mandatory work-related in-home activities below the average duration have a higher likelihood of performing work-related out-of-home activities. This result reveals that those who are working from home are less likely to travel outside for work purposes. One of the interesting findings of the study is that individuals who are involved in online shopping for lower than the average duration have a higher likelihood of traveling out-of-home for regular shopping purposes.

However, this variable reveals significant heterogeneity with a higher value of the standard deviation than the mean value of the random parameter. Therefore, they might also prefer not to perform out-of-home shopping activities. Individuals who perform in-home leisure activities such as relaxing, socializing, watching TV, exercise, hobbies, or games for above average duration are less likely to travel outside for recreation or socializing. This result indicates that safety concerns for the pandemic have forced individuals to do their leisure activities at home rather than traveling outside. On the other hand, individuals performing discretionary activities above the average duration are more likely to travel out-of-home for recreation or socializing.

#### Elasticity Effects

The parameter estimation results in Table 2 do not fully reflect the magnitude of their impact. To determine the magnitude of the impact, the elasticity effects of the variables retained in the final model are estimated (Table 3). The elasticity value for a continuous variable is estimated as the percentage change in the probability of participating in an out-of-home activity as a result of a 1% change in that variable. On the other hand, the elasticity for a dummy variable is estimated by changing the value of the variable to 1 where the value of that subsample of observations is 0. In the case of subsample having a value of 1, elasticity is estimated by changing the value to 0. Finally, the sign of the shifts in the second subsample was reversed, followed by computing the summation of the shifts in the expected aggregate shares of the two subsamples. This gives the effective percentage change in aggregate shares for the total sample resulting from the change in the variable value from 0 to 1 (*4*, *35*).

**Table 3.** Elasticity Effects of the Variables Retained in the Out-of-home Activity Participation Model

Variables	Work-related	Household errands and others	Pick up online orders	Shopping	Recreational/social
Socio-demographic characteristics					
Age 18–34	na	−0.77	−0.7	−0.52	na
Age 35–64	0.78	na	na	na	na
Income > $80,000	0.08	0.33	na	na	0.18
Income ≤ $50,000	na	na	0.36	0.19	na
Gender: male	na	0.39	na	na	na
Full-time worker	0.27	na	na	na	−0.15
Occupation: health	0.07	na	−0.16	na	na
Occupation: management	na	0.17	na	na	na
Occupation: sales	na	0.07	na	na	na
Travel characteristics					
Holds a driver’s license	na	na	1.71	1.17	na
Vehicle ownership	1.26	0.95	0.65	na	na
Travel companionship: family	na	na	0.18	0.09	na
In-home activity duration					
Mandatory duration below average	0.18	na	na	na	na
Online grocery shopping below average	na	na	na	0.74	na
Leisure activity above average	na	na	na	na	−0.56
Discretionary activity above average	na	na	na	na	1.67

*Note:* na = not available.

The analysis shows that individuals aged 35–64 years are 0.78% more likely to participate in work-related out-of-home activities. Being a full-time worker might increase the probability of participating in work-related activities by 0.27% while decreasing the probability of participating in recreational/social activities by 14.97%. The effect of mobility tools such as vehicle ownership and holding a driver’s license shows significant impacts on out-of-home activity participation. A unit percentage increase in household vehicle ownership is likely to increase the likelihood of participating in work-related, household errands, and others, and picking up online orders by 1.26%, 0. 95%, and 0.65%, respectively. Similarly, holding a driver’s license may increase the likelihood of participating in picking up online orders and shopping activities by 1.71% and 1.17%, respectively. The analysis results also show that in-home activity duration has a significant effect on out-of-home activity participation. For example, individuals who perform in-home leisure activities for a longer duration are 0.56% less likely to travel out-of-home for recreational/social activities.

### In-home Activity Duration Model Results

The summary statistics of the variables retained in the in-home activity participation model are represented in Table 4. Table 5 represents the parameter estimation results and the goodness-of-fit measures of the HRPD model of in-home activity duration. The adjusted r-squared, AIC, and BIC values of the model are found to be 0.087, 2,307.87, and 2,410.45, respectively. For comparison, a conventional hazard-based duration (HD) model was developed which gives a lower adjusted r-squared value (0.061), whereas higher AIC (2,363.94) and BIC (2,448.06) values. The HRPD model, therefore, outperforms the HD model and is considered the final model for in-home activity duration.

**Table 4.** Summary Statistics of the Variables Retained in In-home Activity Duration Model

Variables	Description	Mean or percentage	Standard deviation
Socio-demographic characteristics			
Age 30 or more (reference)	Individual’s age is 30 or more (dummy)	89.63%	na
Age 18–29	Individual’s age is 18–29 (dummy)	10.37%	na
Income > $50,000 (reference)	Individual’s yearly income is > $50,000 (dummy)	69.36%	na
Income ≤ $50,000	Individual’s yearly income is ≤ $50,000 (dummy)	30.64%	na
Male (reference)	Individual is male (dummy)	43.31%	na
Female	Individual is female (dummy)	56.69%	na
Out-of-home activity participation			
Age 18–29 × others (reference)	Individual’s age is 18–29 and does not participate in mandatory or discretionary activities (dummy)	97.71%	na
Age 18–29 × mandatory	Individual’s age is 18–29 and participates in mandatory activities (dummy)	1.93%	na
Age 18–29 × discretionary	Individual’s age is 18–29 and participates in discretionary activities (dummy)	0.36%	na
Age 30–44 × other (reference)	Individual’s age is 30–44 and does not participate in mandatory/personal maintenance/leisure activities (dummy)	88.78%	na
Age 30–44 × mandatory	Individual’s age is 30–44 and participates in mandatory activities (dummy)	2.77%	na
Age 30–44 × personal maintenance	Individual’s age is 30–44 and participates in personal maintenance activities (dummy)	4.22%	na
Age 30–44 × leisure	Individual’s age is 30–44 and participates in leisure activities (dummy)	4.22%	na
Male × other (reference)	Individual is male does not participate in discretionary/household maintenance activities (dummy)	90.35%	na
Male × discretionary	Individual is male and participates in discretionary activities (dummy)	1.93%	na
Male × household maintenance	Individual is male and participates in household maintenance activities (dummy)	7.72%	na
Female × other (reference)	Individual is female and does not participate in leisure activities (dummy)	89.02%	na
Female × leisure	Individual is female and participates in leisure activities (dummy)	10.98%	na
Occupation: health × other (reference)	Individual is a health care professional and does not participate in mandatory/household/personal maintenance activities (dummy)	95.66%	na
Occupation: health × mandatory	Individual is a health care professional and participates in mandatory activities (dummy)	0.97%	na
Occupation: health × household maintenance	Individual is a health care professional and participates in household maintenance activities (dummy)	1.57%	na
Occupation: health × personal maintenance	Individual is a health care professional and participates in personal maintenance activities (dummy)	1.81%	na
Occupation: education × other (reference)	Individual is a community/government services/law and social/education professional and does not participate in mandatory activities (dummy)	97.35%	na
Occupation: education × mandatory	Individual is a community/government services/law and social/education professional and participates in mandatory activities (dummy)	2.65%	na
Out-of-home activity frequency: work	Frequency of out-of-home work/school-related activities in most recent weekday	0.46	0.87

*Note:* na = not available.

**Table 5.** Parameter Estimation Results of the In-home Activity Duration Model

Variables	Coefficient	*t*-statistic
Constant	5.71	106.22***
Socio-demographic characteristics		
Age 18–29	−0.37	−4.26***
Income ≤ $50,000	−0.09	−1.56
Female	−0.40	−6.42***
Out-of-home activity participation		
Age 18–29 × mandatory	−0.15	−0.73
Age 18–29 × discretionary	−1.65	−3.23***
Age 30–44 × mandatory	0.54	2.07**
Age 30–44 × personal maintenance	−0.84	−4.91***
Age 30–44 × leisure	−0.44	−2.33**
Gender: male × discretionary	−1.71	−4.45***
Gender: male × household maintenance	−0.61	−7.16***
Gender: female × leisure	0.31	3.25***
Occupation: health × mandatory	0.45	1.07
Occupation: health × personal maintenance	−0.29	−1.31
Occupation: health × household maintenance	−0.96	−3.39***
Occupation: education × mandatory	0.61	2.74***
Out-of-home activity freq.: work	−0.05	−1.48
Standard deviation of random parameters		
Age 18–29	0.49	6.25***
Income ≤ $50,000	0.38	8.15***
Age 18–29 × mandatory	1.80	8.39***
Female	0.52	13.98***
Goodness-of-fit measures		
Log likelihood function		−1,131.31
Adjusted r-squared		0.087
AIC		2,307.87
BIC		2,410.46

*Note*: AIC = Akaike Information Criterion; BIC = Bayesian Information Criterion; *** = 1% significance level; ** = 5% significance level.

The model results provide important behavioral insights by revealing the impact of individuals’ socio-demographic characteristics, their interaction with different types of in-home activity duration, and the frequency of out-of-home activity participation on the overall in-home activity duration. Low-income individuals (yearly income ≤ $50,000) are less likely to perform in-home activities for a longer duration. Similarly, younger individuals, aged from 18 to 29, have a lower likelihood of performing in-home activities for a longer period. However, the standard deviation values of these two variables are 0.38 and 0.49, respectively which are greater than their respective mean parameter values of 0.09 and 0.37. Therefore, heterogeneity exists among the sample individuals which indicates that those individuals may opt for working at home in a longer span.

The model results also suggest that young individuals are less likely to take part in work or school-related mandatory activities at home for a longer duration. A statistically significant and higher standard deviation value of the random parameter reveals heterogeneity among the sample individuals, indicating that they may take part in mandatory work for a longer duration. In addition, young individuals are also found to perform discretionary activities (e.g., religious, spiritual, and volunteer activities) for a shorter duration. Middle-aged individuals, aged from 30 to 44, are more likely to perform work-related activities for a longer duration. This result indicates that because of the transition of many workstations and businesses to online during the pandemic, these individuals might be involved in working-from-home for a longer duration. As a result, they are more likely to spend less time in personal maintenance (e.g., personal care, eating or drinking, grooming) and leisure activities (e.g., relaxing, socializing, watching TV, exercise, hobbies, or games). Females are less likely to perform overall in-home activities for a longer duration. However, the higher value of the standard deviation of the random parameter confirms the existence of heterogeneity among females. Females are found to perform leisure activities for longer periods whereas males are less likely to do discretionary and household maintenance activities (e.g., general household activity, house cleaning, caring for household members) for shorter periods. Health care professionals are found to spend more time on mandatory activities at home whereas they are less likely to spend more time on personal and household maintenance activities. Among the other professionals, individuals in community, government, law, social, and educational services are more likely to do mandatory work-from-home for a longer duration. One of the major findings of the study is the impact of out-of-home activity participation on the in-home activity duration. The model results suggest that individuals who are participating in mandatory work-related out-of-home activities have a higher likelihood of performing overall in-home activities for a shorter duration.

## Conclusions

This study investigates participation in out-of-home and in-home activities during COVID-19. One of the key features of this research is to examine the interactions between out-of-home and in-home activities. The motivation for exploring this interaction is to provide an understanding of how individuals are adjusting or replacing their out-of-home activities with in-home activities during the COVID-19 pandemic. Furthermore, this study tests the effects of occupation, travel companionship, mobility tool ownership, and socio-demographic characteristics on daily activities. Data for this study comes from the web-based COVID-19 Survey for assessing Travel impact (COST) that collected data on out-of-home and in-home activity during COVID-19. This study used data from the Okanagan region of British Columbia, Canada. Methodologically, this paper develops the following two models: (1) a RPMNL model for the out-of-home activity participation; and (2) a HRPD model for the in-home activity participation. Both models capture unobserved heterogeneity by assuming a continuous distribution of parameters over the sample individuals.

Overall, the model results provide important behavioral insights into out-of-home activity participation and in-home activity duration immediately after the lockdown of a pandemic. For example, health care workers are more likely to engage in work-related travel during the lockdown as a result of COVID-19. Higher-income individuals are less likely to travel for work than their lower-income counterparts. On the other hand, lower-income individuals have a lower likelihood of being involved with a longer duration of in-home work activities. The model results suggest that significant interactions exist between out-of-home and in-home activities. For example, a higher frequency of out-of-home work-related travel is more likely to result in a shorter duration of in-home work activities. There also exists heterogeneity among the individuals. For instance, a shorter duration of in-home online shopping yields a higher probability for participation in out-of-home shopping activity. However, a large standard deviation of this variable reveals that a negative relationship might exist. Such behavioral insights help to understand potential longer-term behavioral shifts, which consequently might help to develop effective plans and policies such as work-from-home strategies. Furthermore, the behavioral understanding of the immediate response to the COVID-19 pandemic will help to better manage future unprecedented scenarios like COVID-19.

This study has certain limitations. Attitudinal attributes and built-environment variables could not be tested in the model as the survey did not collect the information of the residential and work locations, or attitudes of the respondents during the pandemic. One of the limitations is the smaller sample size. As a result, some important attributes such as the presence of children in the household fails to show statistical significance and is not included in the final model. However, some of the variables are retained in the model despite having a low statistical significance, considering that these variables confirm the a priori hypothesis as well as provide important behavioral insights. Therefore, these variables are retained in the final model with the assumption that they might yield a statistically significant relationship if a larger data set were available. Because of the similar small sample size, we could not thoroughly compare the effects of the same variables for multiple alternatives. Several existing studies have adopted a RPMNL using a relatively small sample size to capture the unobserved heterogeneity (*36*, *37*). In addition, one of the reasons for designing this study to capture unobserved heterogeneity using a small sample is a result of the novelty of this pandemic. We found this to be a gap and wanted to provide reasonable insights at the earliest timeframe. Another method to capture unobserved heterogeneity could have been the latent segmentation-based MNL (LSMNL) model. As a result of the small sample size, model estimation for multiple segments might pose further complexity in the estimation procedure followed by challenges in the interpretation of the coefficients of the variables (*18*). As a result, the LSMNL model was not considered in this study.

Most of the variables were tested for (n-1) choice scenarios. However, because of the lower statistical significance and not confirming the a priori hypotheses, these variables could not be retained in the final model. As a result, this study could not provide comparative behavioral insights into individuals’ participation in different activities. Another limitation of the study is that the respondents were not randomly selected. However, after the data collection, an extensive validation exercise is performed, and the validation results suggest that the survey data reasonably represents the population of the Okanagan region. Sample weighting to match the population is common in descriptive analysis but becomes more complicated when applied to model estimation. If a random sample is used, reweighting is not mandatory. However, convenience sampling often requires weighting. In this paper, a convenience sampling technique is adopted; the sample is biased and, therefore, requires weighting. One of the main reasons for applying weights is to address heteroskedasticity. But weighted estimation can often lead to less efficient estimators (*38*). Endogenous sampling can be a problem requiring the weighting of the data for model estimation. Another limitation is the adoption of a web-based survey tool to collect data, which could introduce some biased responses as individuals participating in the survey might be more accustomed to technology devices such as smartphones and computers and might be intending to stay at home for a longer duration. To avoid this bias, multiple methods of data collection such as web-based and telephone interviews among others could be adopted. Finally, the findings of the study should be interpreted carefully since the data overrepresents individuals with higher vehicle ownership.

Future research should focus on validating the results using a comprehensive set of data collected over a continuous time frame to find out whether these conclusions will hold in other socio-demographic and geographical contexts. In addition, the data used for analysis might be associated with reporting bias. For example, the survey collected information about the in-home activity participation of the respondents for an entire day, specifically during the most recent weekday. Although the information concerns their recent activities, recalling durations of activities is often less accurate than the actual duration. To tackle such challenges, future data collection efforts should focus on alternative survey methodologies, such as smartphone apps, that could be used to collect duration data.

Furthermore, developing simpler alternative models such as MNL models or modeling in-home and out-of-home activity participation within a nested structure, performing in-depth analysis, and comparing the results should be considered for future research. Future research should also focus on jointly modeling the in-home and out-of-home activity participation to account for the unobserved error correlation that might jointly affect the participation in both types of activities.
